# Incidence of home delivery among women living with HIV in Lira, Northern Uganda: a prospective cohort study

**DOI:** 10.1186/s12884-021-04222-5

**Published:** 2021-11-10

**Authors:** Agnes Napyo Kasede, Thorkild Tylleskär, David Mukunya, Josephine Tumuhamye, Grace Ndeezi, Anna Agnes Ojok Arach, Paul Waako, James K. Tumwine

**Affiliations:** 1 Busitema University Faculty of Health Sciences, Department of Public Health, P.O. Box 236, Tororo, Uganda; 2 College of Health Sciences, Department of Paediatrics and Child Health, Makerere University, P.O. Box 7072, Kampala, Uganda; 3 Centre for International Health, Department of Global Public Health and Primary Care, University of Bergen, P.O. Box 7800, 5020 Bergen, Norway; 4 Department of Nursing and Midwifery, Lira University, P. O. Box 1035, Lira, Uganda

**Keywords:** HIV, Women, Home delivery, Facility delivery, PMTCT

## Abstract

**Background:**

Home delivery has been associated with mother-to-child transmission of HIV and remains high among HIV-infected women. Predictors for home delivery in the context of HIV have not been fully studied and understood in Northern Uganda. We therefore aimed to find out the incidence and risk factors for home delivery among women living with HIV in Lira, Northern Uganda.

**Methods:**

This prospective cohort study was conducted between August 2018 and January 2020 in Lira district, Northern Uganda. A total of 505 HIV infected women receiving antenatal care at Lira regional referral hospital were enrolled consecutively and followed up at delivery. We used a structured questionnaire to obtain data on exposures which included: socio-demographic, reproductive-related and HIV-related characteristics. Data was analysed using Stata version 14.0 (StataCorp, College Station, Texas, U.S.A.). We estimated adjusted risk ratios using Poisson regression models to ascertain risk factors for the outcome of interest which was home delivery (which is delivering an infant outside a health facility setting under the supervision of a non-health worker).

**Results:**

The incidence of home delivery among women living with HIV was 6.9% (95%CI: 4.9–9.5%). Single women were more likely to deliver at home (adjusted risk ratio = 4.27, 95%CI: 1.66–11). Women whose labour started in the night (night time onset of labour ARR = 0.39, 95%CI: 0.18–0.86) and those that were adherent to their ART (ARR = 0.33, 95%CI: 0.13–0.86) were less likely to deliver at home.

**Conclusion:**

Home delivery remains high among women living with HIV especially those that do not have a partner. We recommend intensified counselling on birth planning and preparedness in the context of HIV and PMTCT especially for women who are: separated, divorced, widowed or never married and those that are not adherent to their ART.

**Supplementary Information:**

The online version contains supplementary material available at 10.1186/s12884-021-04222-5.

## Background

Facility delivery is recommended in the context of HIV to reduce on the risk of mother to child transmission of HIV (MTCT) [1, 2]. Home delivery has been associated with MTCT of HIV [3, 4] because the risk of transmission is reduced when the deliveries are attended to by skilled birth attendants in health institutions. Furthermore, delivering from home deprives an HIV infected woman of prevention of mother-to-child transmission of HIV (PMTCT) interventions during and immediately after labour and delivery which include: receiving ARV prophylaxis for the baby, emergency caesarean section when required, safe delivery practices and use of standard infection prevention practices. HIV infected women who deliver outside a hospital setting are therefore likely to suffer complications resulting into vertical HIV transmission, maternal and (or) infant death [4]. Facility delivery is therefore essential for HIV-infected women and healthcare workers must accentuate its importance during antenatal care.

In addition, home delivery has been found to rank highly among predictors of maternal and neonatal mortality [4–6]. Skilled care, attendance in a hospital during the antenatal period and child birth are key in facilitating appropriate referral in case of obstetric complications that can potentially lead to maternal or neonatal mortality. The maternal mortality rate in Uganda is 345 per 100,000 live births [7]. In 2017 alone 6000 maternal deaths occurred in Uganda and of these, 110 were HIV-related. The neonatal mortality rate in Uganda is also high at 19 deaths per 1000 live births [7, 8].

Other risk factors that have been associated with home delivery among HIV infected women include non-attendance of antenatal care, cost of delivery, low perceived quality of care, fear of discrimination during facility-based delivery, poor adherence to ART, lack of maternal education and history of previous home delivery [9–17]. Male involvement in maternal and child health care for HIV infected women has been shown to improve utilisation of maternity services like facility-based delivery [18].

Predictors for home delivery among HIV infected and HIV uninfected women may be comparable [10], however some factors are unique to HIV infected women like poor maternal ART adherence [14, 15]. These predictors, especially for women living with HIV, have not been fully studied and understood in Northern Uganda. Furthermore, predictors for home delivery vary across different study contexts. We therefore aimed to find out the incidence and risk factors for home delivery among women living with HIV in Lira, Northern Uganda. These findings helped in the identifying of groups of HIV infected women that are most at risk for home delivery. These groups of women can act as a target group for PMTCT interventions to counteract home delivery.

## Methods

### Study design and setting

This prospective cohort study was conducted between August 2018 and January 2020 at the PMTCT clinic in Lira Regional Referral Hospital (LRRH). LRRH offers free maternity services, annually serves about 5000 antenatal care mothers and conducts approximately 6500 deliveries every year. The PMTCT clinic is an initiative of the Ugandan Ministry of Health where free HIV care and treatment is offered to HIV-infected pregnant women. At the PMTCT clinic, the women receive their antenatal and routine HIV care till the time of delivery. When their expected date of delivery has approached, these women are free to deliver their infants at any health facility of their choice, especially one that is nearest to where they reside. However, the nevirapine syrup for the infant’s prophylaxis can only be provided at the clinic where each woman is registered for her HIV care. The reason for this is to assess, weigh and classify the baby as ‘high risk’ or ‘not high risk’ and to determine the dosage and duration of prophylaxis. After delivery, HIV infected women continue to attend the PMTCT clinic until the infant is 6 weeks old at which point the mother-infant pair is discharged to the mother-baby care point (also known as the early infant diagnosis clinic) for further management. These women have to attend several other clinics during pregnancy and after child birth such as: early infant diagnosis, postnatal, immunisation and family planning clinics. All these clinics are independent of each other and of the PMTCT clinic in terms of structural location.

### Participants and procedures

**Fig. 1:**
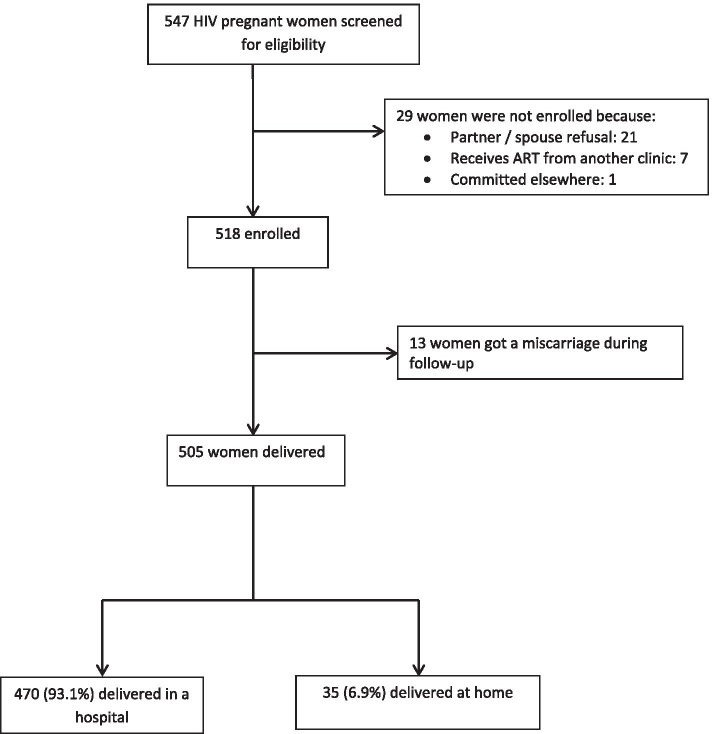
Study flow chart

HIV infected women with a gestational age of 20 weeks or more and receiving antenatal care at LRRH were consented, consecutively enrolled and interviewed on socio demographic characteristics as well as HIV-related information like antiretroviral regimen, duration and a viral load test done during pregnancy. The gestational age and expected date of delivery were estimated using a gestational wheel if the mother had a recollect of the date of the first day of the last normal menstrual period, the palpation method and an ultra sound scan report if the mother had one. They were then followed up with a telephone interview around the time of delivery. At this point, women were interviewed on circumstances surrounding labour and delivery like time of onset of labour, type of delivery, place of delivery, person who supervised the delivery, maternal ART adherence. If the mother had not delivered yet at the time of the interview, another telephone interview was scheduled. Five hundred and five (505) HIV infected pregnant women were included in the final analysis because they had the complete data required (Fig. 1).

Data was collected by trained research assistants who are experienced midwives as well as fluent in Lango and English. Participants were requested to avail their functional telephone contacts or that of a trusted person to minimize loss to follow-up. The research team also documented detailed mapping for each participant’s physical address. In case all the participant’s telephone contacts were unavailable, a home visit was done only if the participant had consented to it at enrolment.

### Sample size estimation

A total of 505 HIV infected pregnant women were enrolled in the study. This sample size for detecting a difference between two independent proportions was calculated using STATA version 14.0 (StataCorp; College Station, TX, USA) assuming 80% power, 95% confidence interval (CI) and 5% precision. We also assumed that of the HIV infected women who delivered at home, 51% were married [19] and that 24.6% were single [15]. The total sample size was then 455 women. After accounting for a non-response of 10% our final sample size was 505 HIV infected women.

### Measurement of variables

The interviews were conducted in *Lango* (the language predominantly spoken in the study setting) and English by trained study staff using a structured questionnaire (this has been provided as a supplementary file). The questionnaires were translated into Lango and back translated into English to ensure consistency in interpretation of information. This translation process was conducted by the Lango translation board which is a certified body in Lango sub-region. Marital status was categorised into married and single. Those who were married or cohabiting were combined into one group and labelled “married”. Those who were separated, divorced, widowed or not married were combined into one group and labelled “single”. We created a composite index of wealth (socio-economic status) by using the principle component analysis on house ownership, availability of electricity in the house, source of drinking water and fuel used for cooking (PCA) [20]. Scores were obtained and categorized into five groups (quintiles) ranging from the poorest to the wealthiest. Women whose labour started between 0600 h to 1859 h (Ugandan time) were all categorised and labelled as “day-time onset of labour” and for those whose labour started from 1900 h to 0559 h were categorised and labelled “night-time onset of labour”. During the follow-up at the time of delivery, for the measurement of maternal ART adherence, we asked the mother, “In the past week, did you miss taking any dose of your medication?” This was a “yes” or “no” response. If the mother answered “yes” she was considered “non-adherent”. The outcome of this study was “home delivery”. Women who delivered in any type of health care setting like national referral hospital, regional referral hospital, public health centre or private clinic were all categorised and considered to have delivered in a “hospital setting”. Those that had delivered at the traditional birth attendant, home or on the road side were all categorised and considered to have delivered in a “non-hospital setting” which we refer to as “home delivery” in the rest of the text for comparability purposes.

### Data analysis and management

We collected data using pretested structured questionnaires. Data was entered into Epi data (www.epidata.dk, version 4.4.3.1) and then exported to Stata version 14.0 (StataCorp, College Station, Texas, U.S.A.) for analysis. Continuous data that was normally distributed was summarised into means and corresponding standard deviations. Frequencies and proportions we calculated for categorical variables. The incidence of home delivery was estimated by dividing the number of women that delivered at home divided by all those who were assessed women, expressed as a percentage and its confidence limits calculated using the exact method. Poisson regression analysis was used for bivariate and multivariate analyses [21]. All variables that had a *p* value < 0.25 at bivariate level and those of biological plausibility like age were collectively put into the initial multivariable model. Then those variables with *p* < 0.1 and those of biological plausibility were put in the second multivariable model while controlling for confounding. We used the likelihood ratio test to check if there was a significant difference between the initial and second models. If there was no difference, we adapted the initial model. We estimated unadjusted and adjusted risk ratios with their corresponding 95% confidence intervals.

## Results

### Socio-demographics

The mean age for the women was 30 years (standard deviation (SD) 5.2). About half of these mothers were 30 years or more (49%) and had attained at least 6 years of schooling (49.9%). The majority were married (93.5%) and unemployed (60.8%). The incidence of home delivery in our cohort was 6.9% (95%CI: 4.9–9.5%). (Table 1).

**Table 1:** Socio-demographic characteristics

	Total Deliveries	Health facility delivery	Home delivery
	(***N*** = 505)	(***N*** = 470)	(***N*** = 35)
Characteristics of mothers	n (%)	n (%)	n (%)
**Socio-demographic**			
**Age**			
≤ 20 years	30 (6.0)	29 (6.2)	1 (2.9)
21–29 years	227 (45.0)	215 (45.7)	12 (34.2)
≥30 years	248 (49.0)	226 (48.1)	22 (62.9)
**Education**			
0–6 years	252 (49.9)	229 (48.7)	23 (65.7)
7–10 years	174 (34.5)	163 (34.7)	11 (31.4)
11–13 years	52 (10.3)	51 (10.9)	1 (2.9)
≥14 years	27 (5.3)	27 (5.7)	0 (0.0)
**Marital status**			
Married	472 (93.5)	444 (94.5)	28 (80.0)
Single	33 (6.5)	26 (5.5)	7 (20.0)
**Employment status**			
Employed	198 (39.2)	187 (39.8)	11 (31.4)
Not employed	307 (60.8)	283 (60.2)	24 (68.6)
**Religious affiliation**			
Christian	487 (96.4)	453 (96.4)	34 (97.1)
Moslem	18 (3.6)	17 (3.6)	1 (2.9)
**Ethnic group**			
Langi	458 (90.7)	425 (90.4)	33 (94.3)
Other	47 (9.3)	45 (9.6)	2 (5.7)
**Socioeconomic index**			
Group 1 (poorest)	107 (21.2)	100 (21.3)	7 (20.0)
Group 2	97 (19.2)	83 (17.7)	14 (40.0)
Group 3	130 (25.7)	121 (25.7)	9 (25.7)
Group 4	82 (16.3)	79 (16.8)	3 (8.6)
Group 5 (wealthiest)	89 (17.6)	87 (18.5)	2 (5.7)

### HIV and reproductive-related characteristics

Most of the women (97%) had disclosed their HIV status and were taking an efavirenz-based ART regimen (89.7%) which was also a first-line regimen. More than half of them (55.5%) had a viral load count of < 50 copies/ml during pregnancy. Majority of these mothers (72.9%) had been pregnant at least four times and had a gestational age of 20–27 weeks pregnant (52.1%) at the time of recruitment. A considerable proportion of these women had a night time onset of labour (54.3%) and gave birth by spontaneous vaginal delivery (87.1%). More than half were adherent to their ART (69.7%) (Table 2).

**Table 2:** Other characteristics

	Total Births	Health Facility Births	Home Births
	**(N = 505)**	**(N = 470)**	**(N = 35)**
**Characteristics of mothers**	**n (%)**	**n (%)**	**n (%)**
**HIV-related**			
**HIV status disclosure**			
Disclosed	490 (97.0)	458 (97.5)	32 (91.4)
Not disclosed	15 (3.0)	12 (2.5)	3 (8.6)
**Antiretroviral treatment**			
Efavirenz-based	453 (89.7)	423 (90.0)	30 (85.7)
Nevirapine-based	43 (8.5)	38 (8.1)	5 (14.3)
Protease inhibitor-based	9 (1.8)	9 (1.9)	0 (0.0)
**Viral load count**			
< 50 cps/ml	280 (55.5)	259 (55.1)	21 (60.1)
50–400 cps/ml	79 (15.6)	73 (15.5)	6 (17.1)
401–499 cps/ml	12 (2.4)	12 (2.6)	0 (0)
> 1000 cps/ml	32 (6.3)	30 (6.4)	2 (5.7)
Missing Viral load result	102 (20.2)	96 (20.4)	6 (17.1)
**Duration of antiretroviral treatment**			
≤ 6 months	95 (18.8)	90 (19.2)	5 (14.3)
7–30 months	109 (21.5)	104 (22.1)	5 (14.3)
31–119 months	267 (52.8)	245 (52.1)	22 (62.8)
≥ 120 months	34 (6.7)	31 (6.6)	3 (8.6)
**Reproductive-related**			
**Baseline**			
**Parity**			
1–4	368 (72.9)	349 (74.3)	19 (54.3)
5–9	137 (27.1)	121 (25.7)	16 (45.7)
**Gestational age (in weeks)**			
20–27	263 (52.1)	244 (51.9)	19 (54.3)
28–35	174 (34.4)	163 (34.7)	11 (31.4)
≥ 36	68 (13.5)	63 (13.4)	5 (14.3)
**Accompanied to antenatal care**			
Not accompanied	453 (89.7)	423 (90.0)	30 (85.7)
Accompanied	52 (10.3)	47 (10.0)	5 (14.3)
**Type of contraceptive used**			
None or “safe days”	258 (51.1)	237 (50.4)	21 (60.0)
Effective contraception	247 (48.9)	233 (49.6)	14 (40.0)
**Intention to have baby**			
No	204 (40.4)	183 (38.9)	21 (60.0)
Yes	301 (59.6)	287 (61.1)	14 (40.0)
**At Birth / Delivery**			
**Type of Delivery**			
Spontaneous vaginal delivery	440 (87.1)	405 (86.2)	35 (100.0)
Caesarean Section delivery	65 (12.9)	65 (13.8)	0 (0.0)
**Time of onset of labour**			
Day time	231 (45.7)	219 (46.6)	12 (34.3)
Night time	274 (54.3)	251 (53.4)	23 (65.7)
**Person who supervised the delivery**			
Health worker	470 (93.0)	463 (98.5)	7 (20.0)
Non-health worker	35 (7.0)	7 (1.5)	28 (80.0)
**Person escorting during delivery**			
Mother	93 (18.4)	89 (18.9)	4 (11.4)
Husband	125 (24.7)	111 (23.6)	14 (40.0)
Mother-in-law	78 (15.5)	72 (15.3)	6 (17.1)
Sibling	52 (10.3)	49 (10.4)	3 (8.6)
Other	157 (31.1)	149 (31.7)	8 (22.9)
**Mother’s adherence to antiretroviral drugs in the past 7 days**			
Did not adhere	153 (30.3)	148 (31.5)	5 (14.3)
Adhered	352 (69.7)	322 (68.5)	30 (85.7)

### Risk factors for home delivery

Single women were more likely to deliver at home (Adjusted Risk Ratio (ARR) = 4.27, 95%CI: 1.66–11) when compared with their married counterparts. HIV infected pregnant women whose labour started in the night time were less likely to deliver at home (night time onset of labour ARR = 0.39, 95%CI: 0.18–0.86) when compared to those whose labour started in the day time. Women who were adherent to their ART were less likely to deliver at home (ARR = 0.33, 95%CI: 0.13–0.86) when compared with those that did not adhere to their treatment (Table 3).

**Table 3:** Risk factors for home delivery among women living with HIV

Variables	Crude IRR	Adjusted IRR
	**(95% CI)**	**(95% CI)**
**Age**		
≤ 20 years	0.73 (0.1–5.44)	0.43 (0.14–1.4)
21–29 years	0.51 (0.23–1.17)	0.56 (0.26–1.2)
≥ 30 years	1	1
**Marital status**		
Married	1	1
Single	2.92 (1.02–8.38)	**4.27 (1.66–11)**
**Socioeconomic index**		
Group 1 (poorest)	1	1
Group 2	2.24 (0.83–6.07)	2.2 (0.85–5.7)
Group 3	1.36 (0.46–4.04)	1.25 (0.46–3.38)
Group 4	0.49 (0.12–1.98)	0.45 (0.11–1.88)
Group 5 (wealthiest)	0.51 (0.1–2.52)	0.56 (0.12–2.69)
**Maternal adherence to ART**		
Did not adhere	1	**1**
Adhered	0.38 (0.13–1.1)	**0.33 (0.13–0.86)**
**Time of onset of labour**		
Day time	1	**1**
Night time	0.42 (0.18–0.94)	**0.39 (0.18–0.86)**

## Discussion

We found a high incidence of home delivery in our study. One study [22] done in Northern Uganda, a context similar to that in our study found that rates of home delivery in the community or general population were higher than that found in our study among HIV infected women. HIV infected women interface with the health care system much more often than their HIV negative counterparts and therefore understand the benefits of health facility delivery especially for the HIV-free survival of their baby hence are most likely to deliver in the hospital that HIV negative women. Various studies have found slightly higher rates of home delivery among HIV infected women. Studies done in Kenya [15], Zimbabwe [10], Malawi [14], South Africa [19] and Nigeria [16] all report higher rates of home delivery among HIV infected women than that in our study. All these studies were conducted in different settings like the community [19] and different types of health facilities like religious based hospitals [14] or public health facilities [15]. The diversity in the settings and study designs employed within the various studies could explain the difference in the rates of home delivery. Furthermore, LRRH and all other Ugandan public health facilities offer free maternity care and delivery services and this could explain the low rates of home delivery among HIV infected women in our study setting.

We also found in our study that single women (separated, divorced, widowed or never married) were more likely to deliver at home. Similar evidence has been found in studies done in South Africa [19], Zambia [23], and Nigeria [24]. Male involvement in maternal and child health care services for HIV infected women improves utilization of these services [7, 9, 18]. Single women lack the social support of a spouse, partner or extended family (in-laws). Social support during pregnancy plays a role in reducing stigma as well emotional and physical stress resulting from pregnancy. Partner support also helps the mother in making the decision to deliver at a health facility. Single women are therefore more likely not to deliver at the hospital because of lack of this social or partner support.

It is surprising to note that women whose labour stated at night were less likely to deliver at home. One study done in Malawi [25] found the contrary. In our study context, culturally, pregnant women approaching the end of the gestational period have to stay with their in-laws (mother-in-law, sister-in-law, grandmother etc). Women living with HIV do understand the risks associated with night-time onset of labour especially home delivery. Due to the extensive support that these women get from the family while pregnant, they are able to make it to the health facility to deliver even if their labour starts at night. More qualitative studies can be done to gain an in-depth understanding of this finding.

This study also demonstrates that women who were adherent to their ART were less likely to deliver at home. A number of studies have shown similar evidence. Studies done in Malawi [14], Zambia [13], Kenya [15, 26] found that poor ART adherence was associated with home delivery. Being adherent to ART is a result achieved from regular interaction with the health care system. These women are able to have regular clinic appointments for their HIV care and other integrated HIV services like counselling on birth planning and preparedness. Women adherent to their ART do understand the importance of health facility delivery in PMTCT and are therefore most likely to deliver in the hospital and not at home.

### Strengths and limitations

This study did have some limitations. Our study was done in a hospital setting therefore findings of this study may only be generalizable to our study context and those similar to it. We never collected information on antenatal care attendance and therefore never included this in our analyses. However, the methodological design of this study in itself is strength because it establishes causality between the covariates and outcome of home delivery. Most of the studies done in this area of inquiry have been cross-sectional in nature and establish only associations.

## Conclusion

Home delivery remains high among women living with HIV especially those that do not have a partner. We recommend intensified counselling on birth planning and preparedness in the context of HIV and PMTCT especially for women who are: separated, divorced, widowed or never married and those that are not adherent to ART.

## Supplementary Information

**Additional file 1.**

## Data Availability

The datasets used and/or analysed during the current study are available from the corresponding author on reasonable request.

## Abbreviations

HIV: Human Immunodeficiency Virus
ART: Antiretroviral therapy
PMTCT: Prevention of mother-to-child transmission of HIV
LRRH: Lira regional referral hospital
CI: Confidence interval
ARR: Adjusted risk ratio
