# Supplementary material for: Incidence of home delivery among women living with HIV in Lira, Northern Uganda: a prospective cohort study
Source: BMC Pregnancy Childbirth. 2021 Nov 10;21:763. doi: 10.1186/s12884-021-04222-5 (PMC8579617; doi:10.1186/s12884-021-04222-5)
Supplement: Supplementary file 1 — Additional file 1. [file 12884_2021_4222_MOESM1_ESM.pdf]

Title: Incidence of home delivery among women living with HIV in Lira, Northern Uganda: A prospective cohort study

Authors:

Agnes Napyo Kasede[1,2,3*], Thorkild Tylleskär[3], David Mukunya[3], Josephine Tumuhamye[3], Grace Ndeezi[2], Anna Agnes Ojok Arach[2,4], Paul Waako[1], James K. Tumwine[2].

Affiliations:

1. Busitema University Faculty of Health Sciences, Department of Public Health, P.O. Box 236, Tororo, Uganda; 2. Makerere University, College of Health Sciences, Department of Paediatrics and Child Health, P.O. Box 7072, Kampala, Uganda; 3. University of Bergen, Centre for International Health, Department of Global Public Health and Primary Care, P.O. Box 7800, 5020 Bergen, Norway; 4. Lira University, Department of Nursing and Midwifery, P. O. Box 1035, Lira, Uganda.

***Correspondence:** napyoagnes@gmail.com

Data collection tool: Questionnaire

AT ENROLLMENT

Demographic data

1. Questionnaire/ID number________________
2. Telephone contact(s)___________________________
3. Age in years: _________________________
4. Occupation:________________________
5. Religion: ______________________________
6. Tribe: __________________________________
7. Address / Residence ____________________________

   District: _________________ Sub-county: _________________ Parish: _____________

   Village: _________________________
8. How far is it from home to this hospital: 1).Above 30 km_________,2)20 – 29 km_______, 3)10 – 19 km________, 4)0 – 9 km_____________
9. What mode of transport did you use to come to the hospital?

   1) Motor bike______________2) Bicycle________________ 3) Motor car ____________

   4) Walking _________________ 5) Other ________________

10. How long did it take you to get to the hospital?

    1) 0 – 30 mins _________________ 2) 30 mins – 1 hour_________________

    3) > 1 hour ________________
11. Who has accompanied you for antenatal today?

    ____
12. Occupation/Employment status: 1)employed___________

    2)unemployed_______________ 3)self-employed______ 4) Others:_____________-________________
13. Do you own or rent the house you live in?

    1) We own it__________ 2) We rent it _______________ 3) Other ____________________
14. Do you have electricity in the house you are living?

    1) Yes ____________________ 2) No ________________ 3) Other ____________________

15. Do you own land?

    1) Yes ______________________ 2) No __________________________

16. What is the main source of drinking water in your household?

    1) Pond, river or stream ___________
    2) Unprotected natural spring_______
    3) Protected natural spring__________
    4) Rainwater_____________________
    5) Open or unprotected well_________
    6) Covered well___________________
    7) Borehole_______________________
    8) Public tap_______________________
    9) Piped into yard / plot_______________
    10) Piped into dwelling_______________
    11) Bottled water____________________
    12) Other, specify____________________

17. What is the main fuel used for cooking in your household?
    1) Wood_______________
    2) Charcoal ____________
    3) Paraffin/ Kerosene _______
    4) Gas ____________________
    5) Electricity _______________
    6) Other, specify____

18. No. of past pregnancies (Parity)__________________

19. No. of children: 1)Alive ____________________ 2)Dead__________________

20. HIV status of each child: 1)________, 2)________, 3)________4)_________ 5)_______

    6)_______

21. Gestation Period / Weeks of Amenorrhea of current pregnancy __________________

22. Marital Status: 1)Married:______________,2) Cohabiting_________,

    3)Separated:_________, 4)Divorced:____________, 5)Widow:______________

    ____

23. Residence Mapping:

    ____

    ____

    ____

    ____

    ____

24. Did you pay any money to get to the facility? 1)Yes ___________ 2) No_____________
25. If Yes, How much money did you pay to get to the facility? _____________________
26. Who gives you the transport money?

    ____

27. Did fears about how other people (for example, your friends, family, employer, or community) would respond if you tested HIV-positive make you hesitate to get tested?

    1) Yes ________________ 2) No __________________________

28. Have you experienced any of the following feelings because of your HIV status?

    1) I feel ashamed ___________________________

    2) I feel guilty ____________________________________

    3) I blame myself __________________________________

    4) I blame others ___________________________________

    5) I have low self-esteem _____________________________

    6) I feel I should be punished ______________________________

    7) I feel suicidal ____

29. What do you understand by the term 'Exclusive Breastfeeding'?________________

    ____

    ____

    ____

30. Do you intend to breastfeed your baby exclusively when you give birth? 1) Yes ______,

    2) No_____

31. If No, Why?

    ____

    ____

    ____

32. If yes, for how long____
33. Did you want / plan to have this baby that you are carrying? 1) Yes _________2) No

    _____

34. If No,

    Why?____

    ____

    ____

35. Were you using any form of family planning prior to this pregnancy?1) Yes_________ 2)No______
36. If Yes, which type of family planning where you using?

    ____

37. Are you already taking any ARVs? 1)Yes ________________2)No_________________
38. If yes, what regimen? ____
39. For how long have you been taking ARVs (duration in years)? ___________
40. Where do you intend to deliver the baby from? 1) Hospital/Health facility_____________2) Home__________________3)TBA__________________ 4)other (Specify) ______________________
41. Was a Viral load sample taken today? (verify with the lab and document code or patient specific number) Code_____________________________

    1) Yes __________________ 2)No__________________________
42. If no, Why?

    ____

    ____

    ____

    ____
43. Have you disclosed your HIV status to anyone? 1)Yes ___________ 2) No_________
44. If Yes, to who? 1) Husband________ 2)Mother in law _________ 3) Sister ________ 4) Brother ________________5) Other (Specify)__________________________
45. If No, why? ____

____

____

____

At Birth (this is a telephone interview to measure outcome 'facility delivery')

1. Have you given birth yet? 1) Yes ________________ 2) No ___________________
2. If Yes, when ______________________________________
3. If No, when will you give birth (EDD)
   ____
4. What time did the labour start?
   ____
5. Where did you deliver from? 1) Hospital _____________ 2) Home ______________
   3) Traditional Birth Attendant____________ 4) Other (specify)__________________
6. Who supervised the delivery? 1) Health worker (specify cadre) _________________
   2) Mother in law_______________ 3) Aunt_____________ 4) Traditional Birth Attendant _______________ 5) Other (Specify)______________________________
7. Who attended to you during the delivery? 1) Mother __________________________
   2) Husband ______, 3) Mother in law ________ 4) Other (specify) _______________
8. Is the baby alive? 1) Yes_______________ 2) No__________________________
9. Did you give the baby anything to drink before giving breast milk?
   1) Yes __________________________ 2) No __________________________
10. If Yes, Why? ____
    ____
    ____
11. If No, what were the circumstances surrounding the death of the baby?
    ____
    ____
    ____
12. Were you given any medicine by the health worker for the baby?
    1) Yes __________ 2) No_____
13. If No, why?
    ____
    ____
14. If yes, did you give it to the baby? 1)Yes ________________ 2) No______________
15. If you did not give the baby the medicine, what reasons did you have?
    1) Forgot________________________ 2) child refused_______________________
    3) my attendant did not know about my status

________________________________ 4) Other (Specify)

____

16. Are you taking your ARVs? 1)Yes _______________ 2) No ________________

17. If "Yes", how are you taking your ARVs? ___________________________________

____

____

____

18. If No, why?

____

____

____

19. How many doses have you missed in the past week?

____

____
